# Circulating inflammatory cytokines and risk of five cancers: a Mendelian randomization analysis

**DOI:** 10.1186/s12916-021-02193-0

**Published:** 2022-01-11

**Authors:** Emmanouil Bouras, Ville Karhunen, Dipender Gill, Jian Huang, Philip C. Haycock, Marc J. Gunter, Mattias Johansson, Paul Brennan, Tim Key, Sarah J. Lewis, Richard M. Martin, Neil Murphy, Elizabeth A. Platz, Ruth Travis, James Yarmolinsky, Verena Zuber, Paul Martin, Michail Katsoulis, Heinz Freisling, Therese Haugdahl Nøst, Matthias B. Schulze, Laure Dossus, Rayjean J. Hung, Christopher I. Amos, Ari Ahola-Olli, Saranya Palaniswamy, Minna Männikkö, Juha Auvinen, Karl-Heinz Herzig, Sirkka Keinänen-Kiukaanniemi, Terho Lehtimäki, Veikko Salomaa, Olli Raitakari, Marko Salmi, Sirpa Jalkanen, The PRACTICAL consortium, CRUK, CAPS, PEGASUS, Marjo-Riitta Jarvelin, Abbas Dehghan, Konstantinos K. Tsilidis

**Affiliations:** 1 Department of Hygiene and Epidemiology, University of Ioannina School of Medicine, Ioannina, Greece; 2 Department of Epidemiology and Biostatistics, School of Public Health, Imperial College London, St Mary’s Campus, London, W2 1PG UK; 3 Center for Life Course Health Research, Faculty of Medicine, University of Oulu, Oulu, Finland; 4 Research Unit of Mathematical Sciences, University of Oulu, Oulu, Finland; 5 Novo Nordisk Research Centre Oxford, Old Road Campus, Oxford, UK; 6 Clinical Pharmacology Group, Pharmacy and Medicines Directorate, St George’s University Hospitals NHS Foundation Trust, London, UK; 7 Clinical Pharmacology and Therapeutics Section, Institute for Infection and Immunity, St George’s, University of London, London, UK; 8 Singapore Institute for Clinical Sciences (SICS), Agency for Science, Technology and Research (A*STAR), Singapore, Singapore; 9 MRC Integrative Epidemiology Unit, University of Bristol, Bristol, UK; 10 Population Health Sciences, Bristol Medical School, University of Bristol, Bristol, UK; 11 Nutrition and Metabolism Branch, International Agency for Research on Cancer, World Health Organization, Lyon, France; 12 Genomics Branch, International Agency for Research on Cancer, World Health Organization, Lyon, France; 13 Cancer Epidemiology Unit, Nuffield Department of Population Health, University of Oxford, Oxford, UK; 14 Department of Population Health Sciences, Bristol Medical School, University of Bristol, Bristol, UK; 15 National Institute for Health Research (NIHR) Bristol Biomedical Research Centre, University Hospitals Bristol NHS Foundation Trust and the University of Bristol, Bristol, UK; 16 Department of Epidemiology, Johns Hopkins Bloomberg School of Public Health, Baltimore, MD USA; 17 School of Biochemistry, University of Bristol, Bristol, UK; 18 Institute of Health Informatics, University College London, London, UK; 19 Health Data Research UK, London, UK; 20 Department of Community Medicine, Faculty of Health Sciences, Arctic University of Norway, Tromsø, Norway; 21 K.G. Jebsen Center for Genetic Epidemiology, Department of Public Health and Nursing, Norwegian University of Science and Technology, Trondheim, Norway; 22 Department of Molecular Epidemiology, German Institute of Human Nutrition Potsdam-Rehbruecke, Nutehtal, Germany; 23 Institute of Nutritional Science, University of Potsdam, Potsdam, Germany; 24 Prosserman Centre for Population Health Research, Lunenfeld-Tanenbaum Research Institute of Sinai Health System, Toronto, Canada; 25 Dalla Lana School of Public Health, University of Toronto, Toronto, Canada; 26 Baylor College of Medicine, Texas, USA; 27 The Stanley Center for Psychiatric Research, Broad Institute of MIT and Harvard, Cambridge, MA USA; 28 Analytical and Translational Genetics Unit, Massachusetts General Hospital, Boston, MA USA; 29 Institute for Molecular Medicine Finland, University of Helsinki, Helsinki, Finland; 30 Northern Finland Birth Cohorts, Infrastructure for Population Studies, Faculty of Medicine, University of Oulu, Oulu, Finland; 31 Center for Life Course Health Research, Faculty of Medicine, University of Oulu, Oulu, Finland; 32 Research Unit of Biomedicine, Medical Research Center, Faculty of Medicine, University of Oulu, and Oulu University Hospital, Oulu, Finland; 33 Department of Clinical Chemistry, Finnish Cardiovascular Research Center - Tampere, Faculty of Medicine and Health Technology, Tampere University, Tampere, Finland; 34 Finnish Institute for Health and Welfare, Helsinki, Finland; 35 Research Centre of Applied and Preventive Cardiovascular Medicine, University of Turku, Turku, Finland; 36 Department of Clinical Physiology and Nuclear Medicine, Turku University Hospital, Turku, Finland; 37 MediCity Research Laboratory, University of Turku, Turku, Finland; 38 Institute of Biomedicine, University of Turku, Turku, Finland; 39 Unit of Primary Care, Oulu University Hospital, Oulu, Finland; 40 UK Dementia Research Institute at Imperial College London, London, UK

**Keywords:** Cytokines, Cancer, Inflammation, Mendelian randomisation

## Abstract

**Background:**

Epidemiological and experimental evidence has linked chronic inflammation to cancer aetiology. It is unclear whether associations for specific inflammatory biomarkers are causal or due to bias. In order to examine whether altered genetically predicted concentration of circulating cytokines are associated with cancer development, we performed a two-sample Mendelian randomisation (MR) analysis.

**Methods:**

Up to 31,112 individuals of European descent were included in genome-wide association study (GWAS) meta-analyses of 47 circulating cytokines. Single nucleotide polymorphisms (SNPs) robustly associated with the cytokines, located in or close to their coding gene (c*is*), were used as instrumental variables. Inverse-variance weighted MR was used as the primary analysis, and the MR assumptions were evaluated in sensitivity and colocalization analyses and a false discovery rate (FDR) correction for multiple comparisons was applied. Corresponding germline GWAS summary data for five cancer outcomes (breast, endometrial, lung, ovarian, and prostate), and their subtypes were selected from the largest cancer-specific GWASs available (cases ranging from 12,906 for endometrial to 133,384 for breast cancer).

**Results:**

There was evidence of inverse associations of macrophage migration inhibitory factor with breast cancer (OR per SD = 0.88, 95% CI 0.83 to 0.94), interleukin-1 receptor antagonist with endometrial cancer (0.86, 0.80 to 0.93), interleukin-18 with lung cancer (0.87, 0.81 to 0.93), and beta-chemokine-RANTES with ovarian cancer (0.70, 0.57 to 0.85) and positive associations of monokine induced by gamma interferon with endometrial cancer (3.73, 1.86 to 7.47) and cutaneous T-cell attracting chemokine with lung cancer (1.51, 1.22 to 1.87). These associations were similar in sensitivity analyses and supported in colocalization analyses.

**Conclusions:**

Our study adds to current knowledge on the role of specific inflammatory biomarker pathways in cancer aetiology. Further validation is needed to assess the potential of these cytokines as pharmacological or lifestyle targets for cancer prevention.

**Supplementary Information:**

The online version contains supplementary material available at 10.1186/s12916-021-02193-0.

## Background

Accumulation of biological evidence has led to the establishment of inflammation as a hallmark of cancer [1]. It has been postulated that a state of low-grade inflammation can increase mutation rates and augment the proliferation of mutated cells by supplying trophic signals [2]. In addition to potential direct cell growth promotion effects, activated inflammatory cells can stimulate reactive oxygen species and the accumulation of reactive nitrogen intermediates in neighbouring cells [1]. These processes may damage DNA and its protein products, directly or indirectly, thus having tumour promoting effects [3, 4].

Observational evidence has shown that diseases characterised by a chronic inflammatory state are associated with an increased risk of several cancers, including lung, prostate and colorectal cancer, while use of nonsteroidal anti-inflammatory drugs such as aspirin, may have a chemopreventive role in several cancers, including colorectal, lung, breast, prostate, endometrial and ovarian [5–8]. Specific circulating inflammatory markers have been linked to cancer development in prospective cohort studies. For example, higher concentrations of circulating C-reactive protein (CRP), a highly sensitive but non-specific marker of elevated inflammatory response, were associated with a higher risk of several cancers, including breast, lung, prostate, ovarian and colorectal cancer [9]. Higher pre-diagnostic concentrations of interleukin 1 alpha (IL-1a), IL-8 and tumour necrosis factor alpha (TNF-A) have been associated with higher risk of ovarian cancer, whereas concentrations of serum amyloid A, soluble tumour necrosis factor receptor-2 (sTNF-RII) and monokine induced by gamma interferon (MIG) have been positively associated with lung cancer risk [10, 11]. If such associations are causal, preventing or intervening on inflammation pathways could be a strategy to reduce cancer risk.

While the reduction of chemokine levels for inhibiting cancer progression has been much discussed in the context of cancer therapy, observational studies linking specific circulating inflammatory cytokine concentrations to cancer risk are sparse, have relatively small sample sizes, and the results from which may be impacted by unmeasured confounding, reverse causation and other biases [12, 13]. An approach to overcome the potential limitations of observational epidemiology and strengthen the evidence for a potential causal role of chronic inflammation on cancer risk is Mendelian randomisation (MR). In MR, germline genetic variants are used as instrumental variables to proxy lifetime exposure for an exposure of interest, in this case circulating cytokines, chemokines, growth factors and interferons (hereafter cytokines). In the present study, MR was used to capture usual cytokine concentration experience over the life course, rather than expression variations such as those resulting from epigenetic alterations. We used genetic variants robustly associated with circulating cytokines to estimate the association of genetically proxied inflammatory cytokine concentrations on risk of breast, endometrial, lung, ovarian and prostate cancer. We used outcome-data from large well-established consortia that were either publicly available or for which we had access to base on active research proposals.

## Methods

**Fig. 1:**
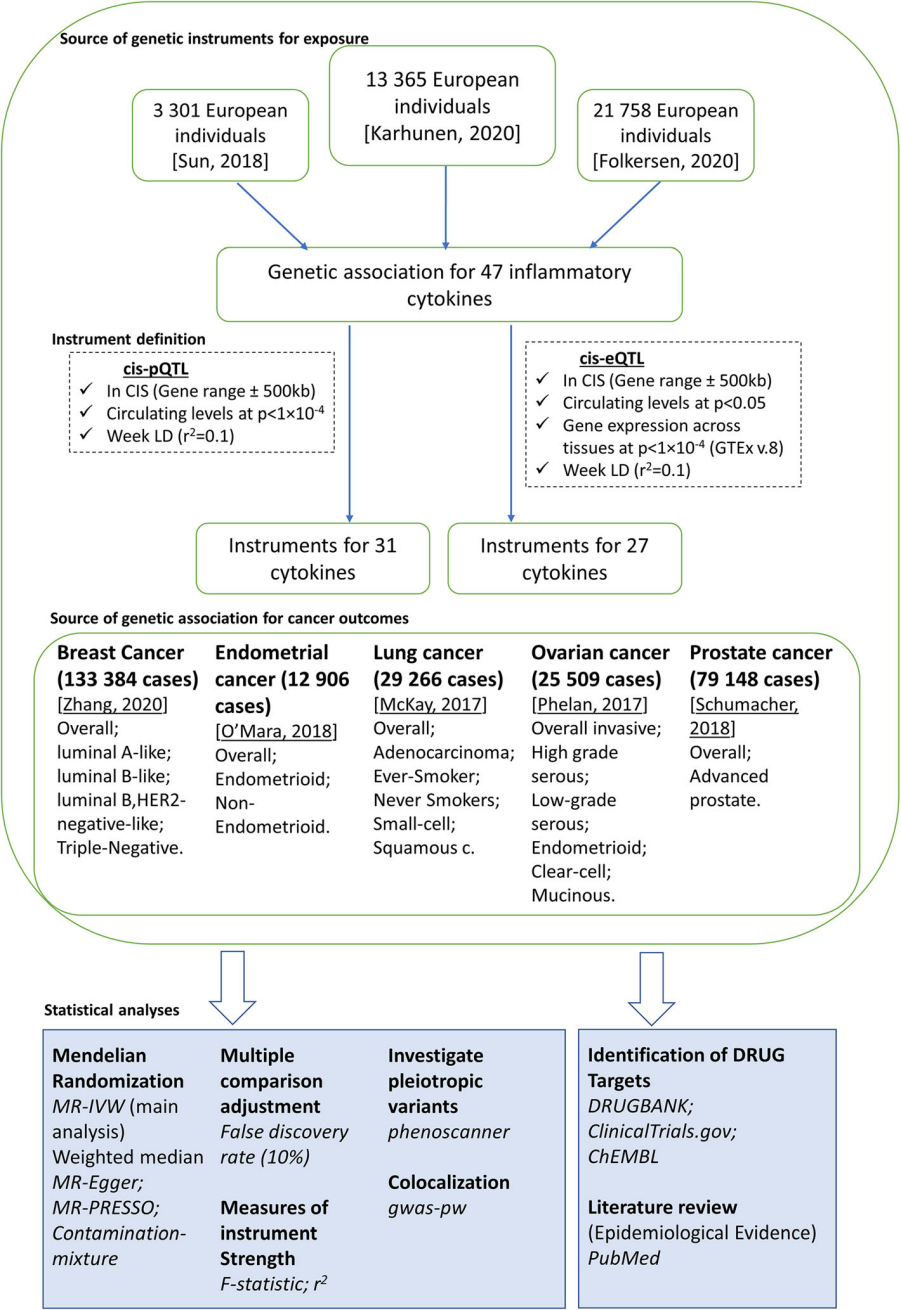
Overview of the analytical plan

An overview of the analytical approach is shown in Fig. 1.

### Cytokine instrument selection

We previously conducted a genome-wide association study (GWAS) of circulating levels of 47 inflammatory cytokines, using samples from up to 13,365 Finnish individuals from the Northern Finland Birth Cohort 1966 (NFBC1966) [14], the Cardiovascular Risk in Young Finns (YFS) study and FINRISK 1997 and 2002 [15, 16]. Publicly available data for several inflammatory cytokines were available from two additional sources: a GWAS of up to 21,758 individuals of European descent from the SCALLOP consortium and a GWAS of up to 3301 individuals of European descent from the INTERVAL study [17, 18]. To obtain the most robust estimates for any given cytokine, the associations of single nucleotide polymorphisms (SNPs) with inflammatory cytokines from these sources were pooled with the Finnish GWAS estimates, when estimates between GWAS correlated well, to include up to 31,112 individuals (ranging from 3301 to 31,112) (Additional file 2: Table S1). Details on the Finnish GWAS and the meta-analysis with the SCALLOP or the INTERVAL GWAS can be found in the Additional file 3.

To minimise the possibility of horizontal pleiotropy, that might occur when a variant influences the cancer outcomes through traits other than the cytokines of interest, we used *cis* instrument definitions. Genetic variants that are located in or close to the coding gene (in *cis*) are naturally more relevant to the expression of that gene (and hence protein concentrations) in comparison to other genes [19]. In addition, trans instruments (obtained from throughout the genome) are less specific to particular cytokines and more likely to be invalid due to pleiotropic functions. We therefore used two distinct cis instrument definitions as described by Karhunen et al. [16]: (i) a cis-protein quantitative trait locus (cis-pQTL) definition, involving cytokines that had genetic variants within the corresponding gene locus (Additional file 2: Tables S1 & S2) extended by 500 kb upstream and downstream, that associated with the circulating cytokine concentrations at *p* < 1 × 10^−4^, which comprised our main analysis, and (ii) a cis-expression quantitative trait locus (cis-eQTL) definition, selecting cytokines with variants within the corresponding gene locus extended by 500 kb upstream and downstream, that associated with both its gene expression aggregated across tissues at *p* < 1 × 10^−4^, and its circulating cytokine concentrations at *p* < 0.05, to replicate the findings from our main analysis and potentially capture additional associations [16]. Cis-eQTL instruments may capture the effects of pQTL instruments via gene expression, but not all pQTLs are represented by eQTLs [20]. Post-transcriptional effects may be represented by cis-pQTL instruments with no corresponding cis-eQTL (e.g. protein degradation, secretion, clearance, etc.) and instrument strength is higher as more pQTLs are available per cytokine. Additionally, by extending the region by 500 kb regulating regions outside the gene may be captured and gain in instrument strength [21]. The gene locations were extracted from human genome build 19 using the University of California Santa Cruz (UCSC) Genome Browser (accessed on 18 June 2019). Gene expression data were obtained from the GTEx Portal (version 8) [20]. Variants with a minor allele frequency (MAF) < 0.05 were excluded. In the context of a cis-region MR, using a very small correlation threshold may result in a loss of causal variants; therefore, clumping was performed using a pairwise linkage disequilibrium (LD) threshold of *r*^2^ < 0. 1[22].

### Outcome data

The Breast Cancer Association Consortium (BCAC) of up to 133,384 women with breast cancer and 113,789 controls of European ancestry was accessed to obtain associations of SNPs with risks of overall breast cancer and five intrinsic-like, molecular subtypes defined by estrogen receptor (ER), progesterone receptor (PR) and human epidermal growth factor receptor (HER) 2 status and tumour grade, namely luminal A-like, luminal B,HER2-negative-like, luminal B-like, HER2-enriched-like and triple-negative (Additional file 2: Tables S3 & S4) [23].

Associations of SNPs with risks of overall endometrial cancer, and endometrioid and non-endometrioid histological sub-types, were obtained from a meta-analysis of 17 studies from the Endometrial Cancer Association Consortium (ECAC), the Epidemiology of Endometrial Cancer Consortium (E2C2) and the UK Biobank, corresponding to a total of 12,906 endometrial cancer cases and 108,979 country-matched controls of European ancestry [24].

Associations of SNPs with risk of overall lung cancer, its predominant histological types (lung adenocarcinoma, small cell carcinoma, squamous cell carcinoma), and associations stratified by smoking behaviour (ever and never smoking) were obtained from the Transdisciplinary Research of Cancer in Lung (TRICL) and the International Lung Cancer Consortium (ILCCO) of 29,266 patients and 56,450 controls of European descent [25].

Associations of SNPs with risk of overall invasive epithelial ovarian cancer and its histological subtypes (high grade serous, low-grade serous, endometrioid, clear-cell and mucinous ovarian cancer) were obtained from the Ovarian Cancer Association Consortium (OCAC) meta-analysis of up to 25,509 epithelial ovarian cancer and 40,941 controls. For overall invasive epithelial ovarian cancer and serous ovarian cancer, we used genetic association estimates from the meta-analysis (MA) that included an additional 31,448 BRCA1 and BRCA2 mutation carriers (including 3887 high grade serous ovarian cancer cases) from the Consortium of Investigators of Modifiers of BRCA1/2 (CIMBA) [26].

Associations of SNPs with risk of overall and advanced prostate cancer (defined as Gleason Score 8+ or death from prostate cancer or metastatic disease (M1) or PSA > 100) were obtained from the Prostate Cancer Association Group to Investigate Cancer-Associated Alterations in the Genome (PRACTICAL) Consortium of 79,148 prostate cancers and 61,106 controls of European descent. Additionally, summary data from two separate analyses were used: estimates describing the association between high vs. low risk and high vs. low or intermediate risk prostate cancer (Additional file 2: Tables S3 & S4) [27].

### Mendelian randomisation analyses

Separate analyses were performed using the two different sets of instruments (cis-pQTL and cis-eQTL) to investigate the associations of genetically proxied circulating cytokine concentrations with the risk of each of the cancer outcomes. When only a single SNP was available to construct the instrumental variable, the ratio of coefficients method was used to obtain MR estimates with first order weights used to generate standard errors. Where more than one SNP was available to construct the instrumental variable for a given cytokine, MR estimates obtained from the individual SNPs within the instrument were pooled using the random-effects inverse-variance weighted (IVW) MR method. To address multiple hypothesis testing, we estimated the false discovery rate (FDR) adjusted *p* values (*q* values), in the main IVW MR analyses, using the sequential *p* value approach proposed by Benjamini and Hochberg [28]. A *q* value not greater than 10% was considered significant. The effect estimates reflect the increase in cancer risk per SD higher in the natural scale of each cytokine.

To measure the strength of the genetic instruments, we calculated the F-statistic and proportion of variance explained (*r*^2^) for each genetic variant based on the circulating protein concentrations [29] (Additional file 2: Table S2). In addition, we computed (post-hoc) the statistical power to detect an odds ratio (OR) of 1.2 per 1-SD increase in circulating cytokine levels, with type I error rate of 0.05 (Additional file 2: Tables S5 & S6) [30]. Since large associations for the genetic propensity for between-individual variability in circulating cytokine concentrations with cancer are generally not anticipated, we used an odds ratio of 1.2 in all power calculations.

The selected genetic variants, to be valid instruments for the MR analysis, must meet the following criteria: (i) they should be strongly associated with the circulating concentrations of the cytokine, (ii) they should be independent of any potential confounding variable of the cytokine-cancer association and (iii) they should affect cancer only through the cytokine being instrumented. The presence of horizontal pleiotropy, that occurs when a variant influences the outcome through other traits (pathways) that bypass the exposure of interest, is the most common reason for violation of the third assumption. We have, in part, adjusted for horizontal pleiotropy “by design” by excluding trans-associated loci. In addition, to evaluate the cytokine specificity, we performed sensitivity analysis either excluding instruments that are associated (*p* < 5 × 10^−8^), in *trans*, with other cytokines, or by multivariate MR [31]. We also used several other sensitivity analyses, namely weighted-median, contamination mixture (ConMix), MR-Egger and MR-PRESSO, though these methods operate best in a polygenic MR analysis framework. Further details on the assumptions of these methods are described in the Additional file 3.

To further assess potential pleiotropic effects for the instruments for which there was evidence of an association from the MR analyses, we used Phenoscanner, a database that includes genotype-phenotype associations [32]. We searched for previously reported associations for any SNP that was included as instrument in our analysis and associations with any secondary phenotypes related to inflammatory traits were considered vertical (in the same pathway from genetic variant to cancer) pleiotropy.

### Systematic review of publicly available databases for medical drugs, observational studies and biological pathways

To provide in silico replication to our findings, we searched DrugBank and ChEMBL using cytokine-specific terms to identify cytokine-related drug targets, and for the identified drugs, detailed searches using conventional drug names or synonyms were performed in clinical trial registries (e.g. *clinicaltrials.gov*) [33, 34]. In addition, to compare the findings of the MR analyses with epidemiological evidence, we searched PubMed for observational studies investigating the association of chronic inflammatory markers in relation to the cancer outcomes of interest, using general MeSH search terms such as “*cytokines*”, “*inflammation*” and “*neoplasms*”. Furthermore, biological pathways that the significant cytokines are involved were identified using the Kyoto Encyclopedia of Genes and Genomes (KEGG) database [35].

### Colocalization analysis

Colocalization analysis evaluates the shared, local genetic architecture between two traits, applying a set of arithmetic operations followed by statistical testing to assess whether or not the observed overlap or spatial proximity is likely to be due to chance [36]. Colocalization analyses are valuable in strengthening the associations observed in MR analysis. They can help identify MR associations that may have arisen due to confounding by LD (i.e. when another genetic variant which is in high LD with a genetic instrument is also associated with the outcome) [37]. We applied a Bayesian framework proposed by Pickrell et al. to detect shared causal variants for the significant associations (FDR ≤ 10%) in the MR analyses [38]. For each of these cytokines-cancer pairs, we used the genomic region extending 25 kb on both sides of the lead cytokine variant. Results with a posterior probability (PP) > 0.8 within the gene locus of the putative causal cytokine of each pair were deemed as evidence for colocalization. We further explored the significant cytokines-cancer associations in colocalization analyses using tissue specific gene expression data (i.e. for a cytokine that was associated with lung cancer, we conducted analyses of pQTL variants vs. lung tissue eQTL data) [39]. Default priors were used in all analyses.

Significant associations (FDR < 10%) that were confirmed in colocalization analyses were replicated using independent outcome data from the UK Biobank [40].

All analyses were performed using R, version 4.0.2 [41].

## Results

### Instrument characteristics and instruments strength

In total, 31 and 27 cytokines (35 unique cytokines) with a median of 5 (IQR 1 to 16) and 2 (IQR 1 to 3) SNPs per instrument were included in the analysis under the cis-pQTL and cis-eQTL definitions, respectively (Table 1 and Additional file 2: Table S2). The respective cytokines were as follows: active plasminogen activator inhibitor-1 (activePAI), beta nerve growth factor (bNGF), cutaneous T-cell attracting chemokine (CTACK), Eotaxin, basic fibroblast growth factor (FGFBasic), growth-regulated oncogene-alpha (GROa), hepatocyte growth factor (HGF), interleukin (IL)-16, IL-18, IL-1a, IL-1ra,IL-2ra, IL-6, IL-7, IL-8, IL-12p70, interferon gamma-induced protein 10 (IP-10), monocyte chemotactic protein-1 (MCP1), monocyte chemotactic protein-3 (MCP3), macrophage colony-stimulating factor (MCSF), macrophage migration inhibitory factor (MIF), monokine induced by interferon-gamma (MIG), macrophage inflammatory protein (MIP)-1a, MIP1b, platelet-derived growth factor BB (PDGFbb), beta-chemokine RANTES (RANTES), stem cell factor (SCF), stem cell growth factor beta (SCGFb), soluble E-selectin (SeSelectin), soluble intercellular adhesion molecule (sICAM) 1, soluble vascular cell adhesion molecule (sVCAM) 1, tumour necrosis factor (TNF)-A, TNF-B, TNF-related apoptosis inducing ligand (TRAIL) and vascular endothelial growth factor (VEGF) (Table 1 and Additional file 2: Tables S2, S5 & S6).

**Table 1:** Instrument strength per cytokine in the cis-pQTL and cis-eQTL analyses

Cytokine	Gene	Gene ensemble	Chr	Start hg19	End hg19	cis-pQTL (***n*** instruments)	pQTL (r^**2**^)	cis-eQTL (***n*** instruments)	eQTL (***r***^**2**^)
activePAI	*SERPINE1*	ENSG00000106366	7	100770370	100782547	1	0.003	–	–
bNGF	*NGF*	ENSG00000134259	1	115828537	115880857	–	–	1	0.002
CTACK	*CCL27*	ENSG00000213927	9	34661893	34662689	3	0.060	2	0.041
Eotaxin	*CCL11*	ENSG00000172156	17	32612687	32615199	6	0.015	4	0.010
FGFBasic	*FGF2*	ENSG00000138685	4	123747863	123819390	–	–	2	0.002
GROa	*CXCL1*	ENSG00000163739	4	74735109	74737019	11	0.272	1	0.127
HGF	*HGF*	ENSG00000019991	7	81331444	81399452	6	0.010	–	–
IL-16	*IL16*	ENSG00000172349	15	81517640	81605104	18	0.037	6	0.031
IL-18	*IL18*	ENSG00000150782	11	112013974	112034840	5	0.051	2	0.024
IL-1a	*IL1A*	ENSG00000115008	2	113531492	113542971	–	–	3	0.003
IL-1ra	*IL1RN*	ENSG00000136689	2	113885138	113891593	18	0.075	2	0.017
IL-2ra	*IL2RA*	ENSG00000134460	10	6052657	6104333	14	0.260	4	0.130
IL-6	*IL6*	ENSG00000136244	7	22766766	22771621	1	0.002	1	0.001
IL-7	*IL7*	ENSG00000104432	8	79645007	79717758	1	0.005	–	–
IL-8	*CXCL8*	ENSG00000169429	4	74606223	74609433	1	0.004	2	0.005
IL-12p70	*IL12A*	ENSG00000168811	3	159706623	159713806	1	0.002	–	–
IL-12p70	*IL12B*	ENSG00000113302	5	158741791	158757481	1	0.002	–	–
IP-10	*CXCL10*	ENSG00000169245	4	76942269	76944689	5	0.020	–	–
MCP-1	*CCL2*	ENSG00000108691	17	32582296	32584220	28	0.006	3	0.001
MCP-3	*CCL7*	ENSG00000108688	17	32597235	32599261	13	0.289	–	–
MCSF	*CSF1*	ENSG00000184371	1	110453233	110473616	13	0.049	3	0.018
MIF	*MIF*	ENSG00000240972	22	24236565	24237409	2	0.019	5	0.020
MIG	*CXCL9*	ENSG00000138755	4	76922623	76928641	1	0.011	2	0.008
MIP1a	*CCL3*	ENSG00000277632	17	34415603	34417506	34	0.217	1	0.059
MIP1b	*CCL4*	ENSG00000275302	17	34431220	34433014	26	0.147	3	0.003
PDGFbb	*PDGFB*	ENSG00000100311	22	39619685	39640957	1	0.001	–	–
RANTES	*CCL5*	ENSG00000271503	17	34198496	34207377	1	0.009	1	0.009
SCF	*KITLG*	ENSG00000049130	12	88886570	88974250	3	0.006	2	0.001
SCGFb	*CLEC11A*	ENSG00000105472	19	51226605	51228981	2	0.016	1	0.004
SeSelectin	*SELE*	ENSG00000007908	1	169691781	169703220	2	0.008	2	0.002
sICAM	*ICAM1*	ENSG00000090339	19	10381517	10397291	25	0.168	2	0.004
sVCAM	*VCAM1*	ENSG00000162692	1	101185196	101204601	1	0.003	1	0.003
TNF-A	*TNF*	ENSG00000232810	6	31543344	31546112	2	0.004	–	–
TNF-B	*LTA*	ENSG00000226979	6	31539876	31542100	–	–	1	0.001
TRAIL	*TNFSF10*	ENSG00000121858	3	172223298	172241297	46	0.027	5	0.006
VEGF	*VEGFA*	ENSG00000112715	6	43737946	43754223	21	0.073	1	0.0004

*activePAI* active plasminogen activator inhibitor-1, *bNGF* beta nerve growth factor, *CTACK* cutaneous T-cell attracting chemokine, *FGFBasic* basic fibroblast growth factor, *GROa* growth-regulated oncogene-alpha, *HGF* hepatocyte growth factor, *IL* interleukin, *ra* receptor antagonist, *IP-10* interferon gamma-induced protein 10, *MCP1* monocyte chemotactic protein-1, *MCP3* monocyte chemotactic protein-3, *MCSF* macrophage colony-stimulating factor, *MIF* macrophage migration inhibitory factor, *MIG* monokine induced by interferon-gamma, *MIP* macrophage inflammatory protein, *PDGFbb* platelet-derived growth factor BB, *SCF* stem cell factor, *SCGFb* stem cell growth factor beta, *SeSelectin* soluble E-selectin, *sICAM* soluble intercellular adhesion molecule, *sVCAM* soluble vascular cell adhesion molecule, *TNF* tumour necrosis factor, *TRAIL* TNF-related apoptosis inducing ligand, *VEGF* vascular endothelial growth factor

The median variance explained (*r*^2^) by the genetic variants, per cytokine, was 1.60% (IQR 0.55 to 6.65%) for the cis-pQTL and 0.51% (IQR 0.20 to 1.90%) for the cis-eQTL (Table 1). The F-statistic for cytokines (averaged per cytokine) was ≥ 10 for 90% of the cytokines in the cis-pQTL analysis (range 4 to 186) and for 67% of the cytokines in the cis-eQTL analysis (range 5 to 1194) (Additional file 2: Table S2). In (post-hoc) power estimations, power ≥ 80% to detect an odds ratio of 1.2 (per SD), assuming a type I error rate of 0.05, was available for 37% (median power 0.51) and 20% (median 0.21) of the analyses using the pQTL and eQTL instrument definitions, respectively.

Using the cis-pQTL instrument definition, 75 of the 1,013 associations (7.4%) that were investigated using MR IVW were nominally significant (*p* < 0.05), while considering an FDR of 10% or less, 12 associations were significant, five of which were replicated (*p* < 0.05) in analyses using the cis-eQTL instrument definition. Regarding these 12 associations, the median number of SNPs per instrument was 11.0 (IQR 5.0 to 18.75) and the median *r*^2^ was 6.6% (IQR 4.9 to 9.96%). The significant (*p* < 0.05) MR IVW estimates using the cis-pQTL instrument definition positively correlated with MR IVW estimates using the cis-eQTL (*r*^2^ = 0.55; Additional file 4: Figure S1).

The FDR significant associations are presented in more detail, by cancer outcome, below.

### Cytokine associations with site-specific cancers using MR

#### Breast cancer

**Fig. 2:**
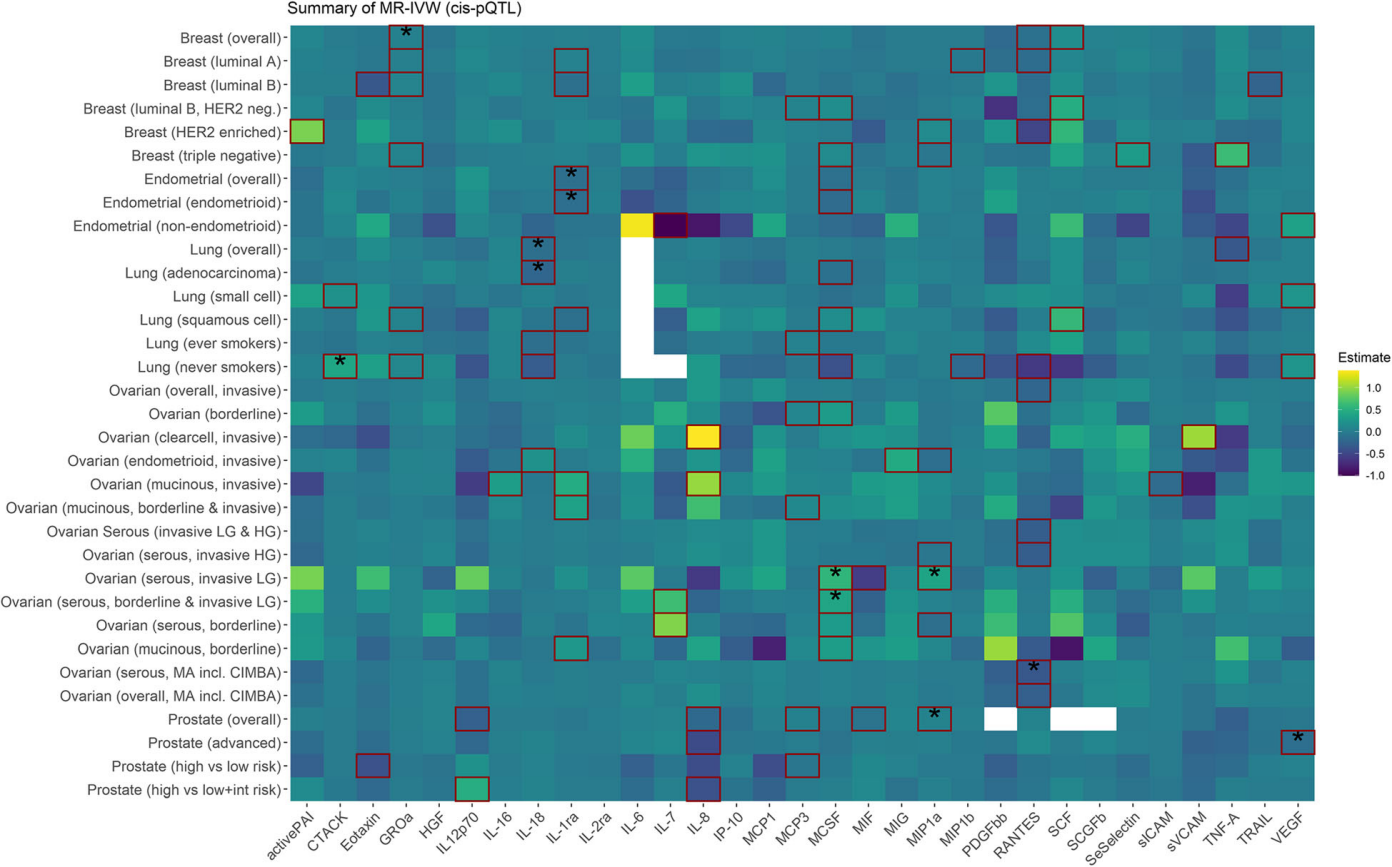
Summary of the MR-IVW results based on the cis-pQTL instrument definition. Squared tiles indicate that the association is nominally significant (*p* < 0.05), and the asterisk denotes that the association was significant when considering multiple comparison correction (FDR ≤ 10%). Colour is scaled based on the MR beta estimates, while associations for which no instrument was available are presented as white tiles

**Fig. 3:**
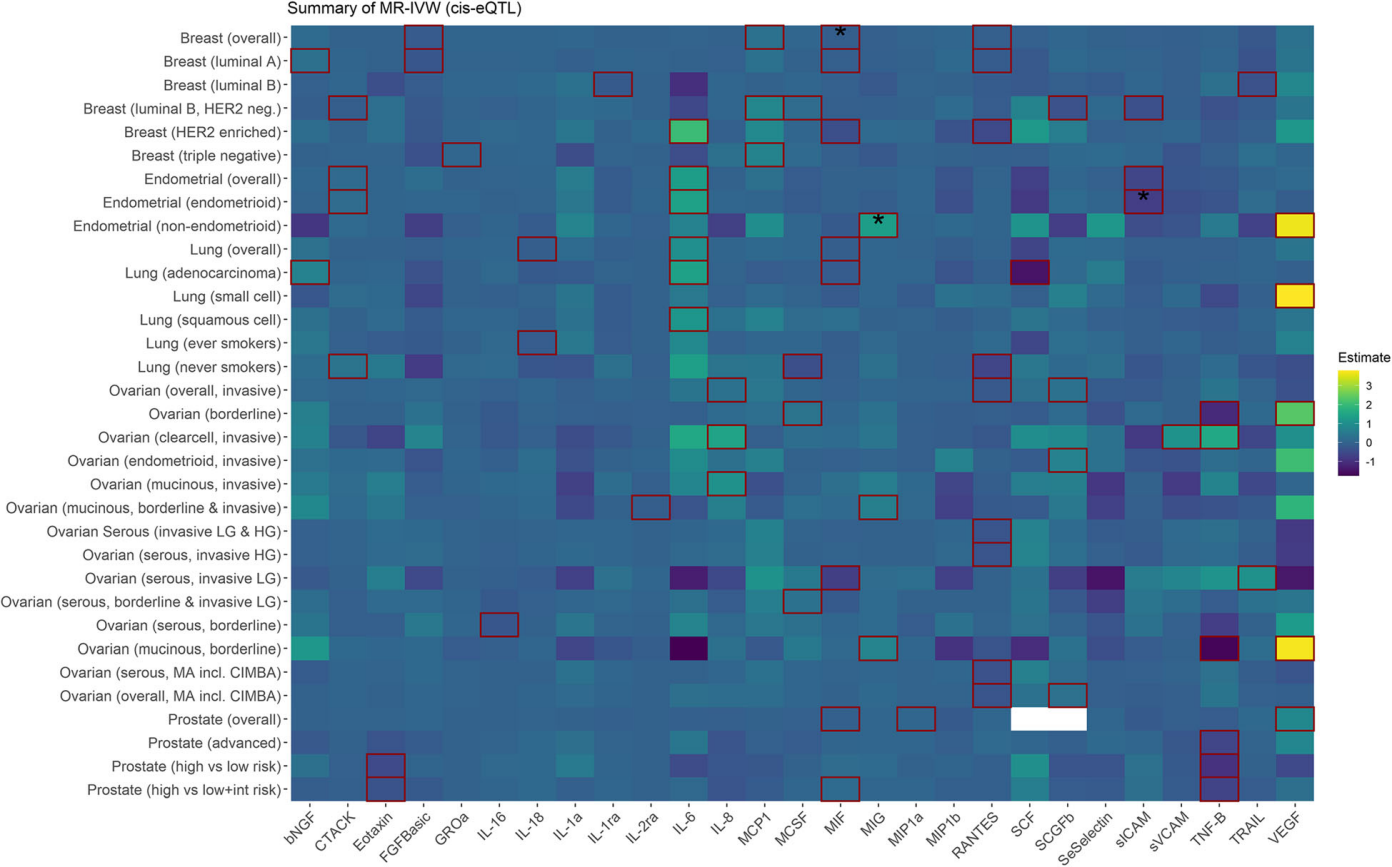
Summary of the MR-IVW results based on the cis-eQTL instrument definition. Squared tiles indicate that the association is nominally significant (*p* < 0.05), and the asterisk denotes that the association was significant when considering multiple comparison correction (FDR ≤ 10%). Colour is scaled based on the MR beta estimates, while associations for which no instrument was available are presented as white tiles

**Fig. 4:**
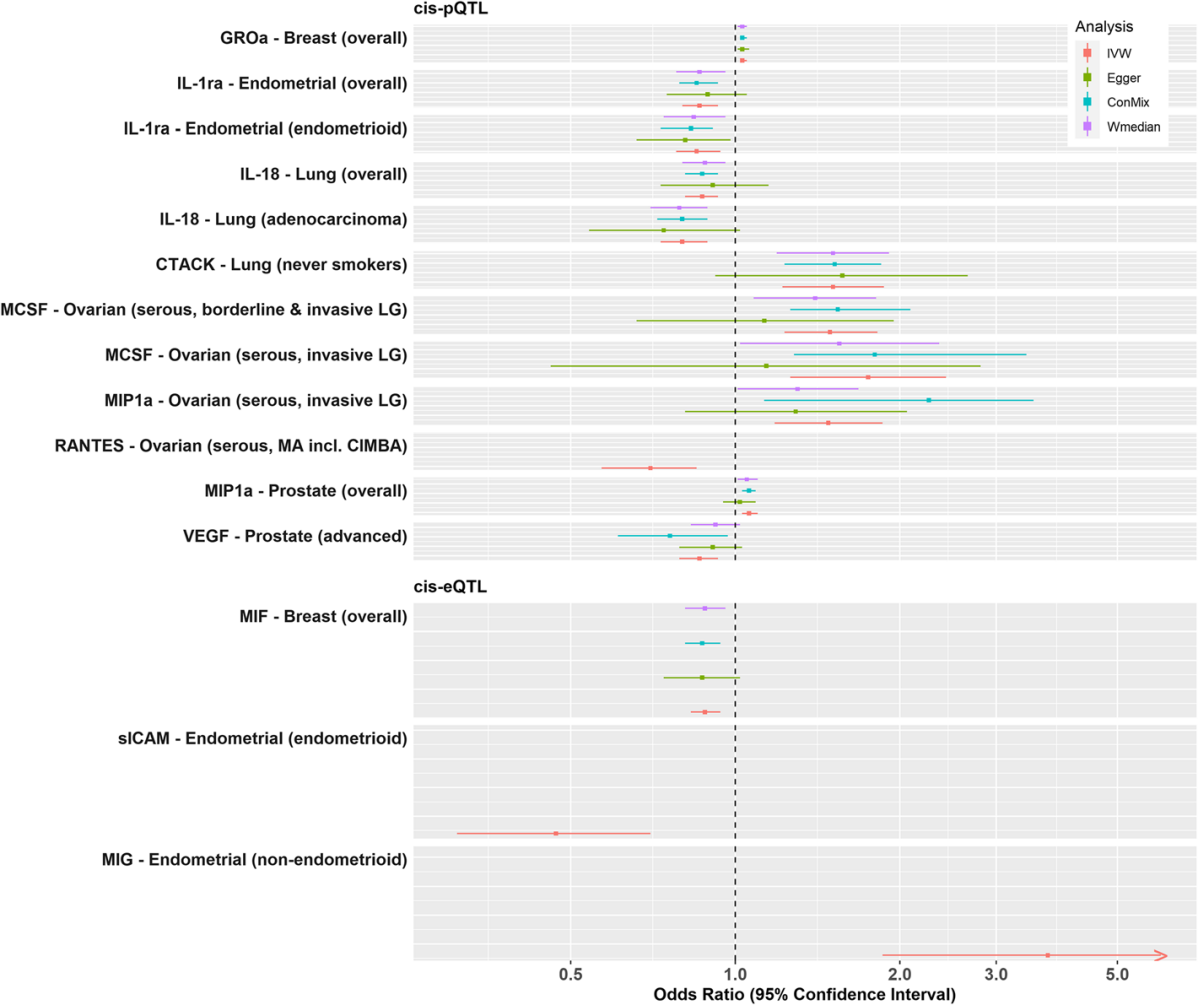
Summary MR-IVW and sensitivity analyses of the associations that were significant when considering multiple comparison correction (FDR ≤ 10%)

Using the cis-pQTL instrument selection criteria, we found evidence of a positive association between genetically proxied concentrations of growth-regulated oncogene-alpha (GROa/*CXCL1*) and overall breast cancer risk (odds ratio [OR], 95% confidence interval [CI] 1.03, 1.02 to 1.05, *p* = 1.09 × 10^−4^), with little evidence of heterogeneity or directional pleiotropy and associations were similar in all the sensitivity analyses (Figs. 2, 3 and 4; Additional file 2: Tables S5 & S6). Using the cis-eQTL criteria and applying a FDR correction (FDR ≤ 10%), an inverse association between genetically proxied circulating concentrations of macrophage migration inhibitory factor (MIF/*MIF*) and overall breast cancer risk (0.88, 0.83 to 0.94, *p* = 1 × 10^−4^) was found, compatible with the results from sensitivity analyses.

#### Endometrial cancer

Genetically proxied concentrations of interleukin 1 receptor antagonist (IL-1ra/*IL1RN*) were inversely associated with overall endometrial cancer risk (0.86, 0.80 to 0.93, *p* = 2.23 × 10^−4^) and the endometrioid subtype (0.85, 0.78 to 0.94; *p* = 7.9 × 10^−4^), and the results were similar in all sensitivity analyses (Figs. 2, 3﻿ and 4; Additional file 2: Tables S5 & S6). Using the eQTL definition and applying an FDR correction (FDR ≤ 10%), a positive association between monokine induced by gamma interferon (MIG/*CXCL9*) and non-endometrioid endometrial cancer risk was observed (3.73, 1.86 to 7.47, *p* = 2 × 10^−4^). We also observed inverse associations between genetically proxied circulating soluble intercellular adhesion molecule 1 (sICAM/*ICAM1*) and endometrioid endometrial cancer risk (0.47, 0.31 to 0.70, *p* = 1.96 × 10^−4^).

#### Lung cancer

Using the cis-pQTL instrument selection criteria, we found a positive association between genetically proxied circulating concentrations of cutaneous T-cell attracting chemokine (CCL27/*CTACK*) and lung cancer risk in never smokers (2355 cases, 1.51, 1.22 to 1.87, *p* = 1.73 × 10^−4^) and inverse associations between genetically proxied interleukin-18 (IL-18/*IL-18*) concentrations and overall lung cancer (0.87, 0.81 to 0.93, *p* = 9.9 × 10^−5^) and lung adenocarcinoma (0.80, 0.73 to 0.89, *p* = 1.41 × 10^−5^) (Figs. 2, 3﻿ and 4; Additional file 2: Tables S5 & S6).

#### Ovarian cancer

Using the cis-pQTL definition, positive associations were observed for genetically proxied macrophage colony-stimulating factor 1 (MCSF/*CSF1*) (1.75, 1.26 to 2.43; *p* = 7.95 × 10^−4^) and macrophage inflammatory protein 1-alpha (MIP1a/*CCL3*) (1.48, 1.18 to 1.86, *p* = 8.19 × 10^−4^) in relation to low-grade serous ovarian cancer (Figs. 2, 3 and 4; Additional file 2: Tables S5 & S6). Furthermore, an inverse association for genetically proxied concentrations of Beta-Chemokine RANTES (RANTES/*CCL5*) in relation to serous ovarian cancer (0.70, 0.57 to 0.85, *p* = 3.8 × 10^−4^) was found, which was based on a single instrumental variable, hence no sensitivity analyses were performed.

#### Prostate cancer

Using the cis-pQTL instrument selection criteria, we found evidence of a positive association between genetically proxied concentrations of MIP1a and overall prostate cancer risk (1.06, 1.03 to 1.1, *p* = 5.62 × 10^−4^) and for an inverse association between genetically proxied vascular endothelial growth factor (VEGF/*VEGFA*) concentrations and risk of advanced prostate cancer (0.86, 0.79 to 0.93; *p* = 2.28 × 10^−4^). The above estimates were similar in all sensitivity analyses (Figs. 2, 3﻿ and 4; Additional file 2: Tables S5 & S6).

### Systematic review of publicly available databases for medical drugs, observational studies and biological pathways

Of the 11 cytokines that showed evidence of a causal link with a cancer, records on past or present clinical drug development programmes were identified for six cytokines (RANTES, MIF, VEGF, IL-1ra, MIP1a, sICAM), four of which on drugs that have already been marketed (MIF, VEGF, IL-1ra and sICAM). Among the indications and associated conditions for these drugs are macular degeneration, skin disorders, cardiovascular disease, multiple sclerosis, cryopyrin-associated periodic syndrome, and as a pain remedy in inflammatory conditions of the joints and against microbial infections. Furthermore, drugs targeting VEGF have been used in several chemotherapy regimens to treat metastatic cancers, including non-squamous non-small cell lung cancer and epithelial ovarian cancer (Additional file 2: Table S7). Epidemiological evidence for the observed associations was available for IL-1ra in relation to endometrial cancer, from a nested case-control study (OR = 1.28 per doubling concentrations; 95% CI 1.06 to 1.54). Regarding the rest of the cytokines for which we found evidence of association from the MR analyses, observational epidemiological evidence was sparse (Additional file 2: Table S8).

Eight of the 11 cytokines that showed evidence of a causal link with a cancer were identified in the KEGG pathway database. The most common across cytokines was the ‘*Cytokine-cytokine receptor interaction*’ pathway, while most of these cytokines were involved in known cancer-related pathways, such as the ‘*MAPK’* (MCSF; VEGF), the ‘*NF-kappa B*’ (GROa; sICAM), the ‘*PI3K-Akt*’ (MCSF; VEGF), the ‘*Ras*’ (MCSF; VEGF), the ‘*HIF-1*’ (VEGF) and the ‘*Toll-like receptor*’ (MIG) signalling pathways among others (Additional file 2: Table S9).

### Colocalization

We found evidence to support the presence of a shared causal variant, using colocalization analysis (posterior probability for shared variant > 0.8) for the associations between MIF and overall breast cancer, IL-1ra and endometrioid endometrial cancer, IL-18 and lung adenocarcinoma, CTACK and lung cancer (in never smokers) and RANTES in relation to serous ovarian cancer (Additional file 2: Table S10). Regional colocalization plots for these associations are provided in Additional file 4: Figure S2.

When tissue specific gene-expression estimates were used, significant results were found for MIF in relation to breast tissue, IL-18 in relation to lung tissue and for MIG expression in the corpus uteri tissue (Additional file 2: Table S10).

### Secondary traits associated with selected instruments and sensitivity analyses

Genetic variants that were used as instruments for specific cytokines were concomitantly associated (in *trans*) with other cytokines (Additional file 2: Table S11a). Sensitivity analysis, excluding these variants, did not materially alter the effect estimates (Additional file 2: Table S12). Several SNPs used as instruments in our analyses have also been associated with other inflammation-related traits, such as white blood cell count, CRP, rheumatoid arthritis and eczema, strengthening their biological relevance as instrumental variables (Additional file 2: Table S11b). Two SNPs, namely *rs868340* (used under the pQTL instrument definition for MIP1a) and *rs281431* (used under the eQTL instrument definition for sICAM), were associated with secondary traits, such as body mass index and height, potentially introducing horizontal pleiotropy. Results were similar in sensitivity analyses, excluding potentially pleiotropic variants (Additional file 2: Table S12a). In addition, when we adjusted for the potential small LD among variants, results were qualitatively the same (Additional file 2: Table S12b).

Only the association of IL-18 in relation to lung cancer was replicated (*p* < 0.05) in the UK Biobank (Additional file 2: Table S13); power though was substantially limited (5%) in all analyses.

## Discussion

We used MR analyses to investigate potential causal links between genetically proxied circulating concentrations of several inflammatory-related cytokines and risk of breast, endometrial, lung, ovarian and prostate cancer. We found an inverse association between genetically proxied concentrations of MIF and breast cancer, a positive association of MIG and an inverse association of IL-1ra with endometrial cancer, a positive association of CTACK and an inverse association of IL-18 with lung cancer and an inverse association of RANTES with epithelial ovarian cancer. These findings were similar among sensitivity analyses and were supported in colocalization analyses. We also found a positive association of GROa with breast cancer, an inverse association of sICAM with endometrial cancer, positive associations of MCSF and MIP1a with epithelial ovarian cancer and a positive association of MIP1a and an inverse association of VEGF with prostate cancer. These findings were similar in sensitivity analyses but were not supported by colocalization analyses.

Previous MR analyses have investigated associations of cytokines with cancer, but in general used trans SNPs in the construction of instrumental variables. One MR study that investigated the effect of 27 cytokines and growth factors on the risk of prostate cancer, drawing IV estimates from a previously published GWAS on 8293 Finnish individuals and the same population that we included in our analysis for prostate cancer, found that higher genetically proxied circulating concentrations of C-C motif chemokine ligand 2 (MCP-1/*CCL2*) were associated with a higher risk of prostate cancer [42]. Another MR study using the same source of instrumental variables investigated 24 cytokines in relation to breast cancer risk and demonstrated positive associations for MCP-1, MIP1b and IL-13 [43]. Such associations were not replicated in our analysis, most likely due to a different instrument definition. Contrary to the previous MR studies, we used a cis definition to instrument the inflammatory biomarkers, selecting variants in close proximity to the encoding gene region, thus reducing the likelihood of horizontal pleiotropy [44]. Furthermore, previous MR studies used publicly available GWAS estimates for cytokines that were adjusted for BMI and these estimates may suffer from collider bias (a variable that is a common effect of two other variables) [45, 46], when cytokine concentrations affect directly BMI levels that has been observed in the literature for some cytokines [15].

We found an inverse association between genetically proxied MIF concentrations and breast cancer risk. MIF is a pro-inflammatory cytokine, aberrantly expressed in many solid tumours, including breast, and it has been shown to promote tumour progression and metastasis [47]. Additionally, due to its functional properties, it has been characterised as a promising target for anti-cancer treatment development [48]. Studies in various breast cancer cell lines and human breast cancer tissue have indicated a potential role in breast cancer invasion and immunomodulation, though its functional role is not fully understood [49]. Overexpression of MIF has also correlated with worse survival in triple-negative breast cancer compared to other hormonal status [50]. Despite findings from several experimental studies suggesting a positive association, our study showed an inverse association for genetically proxied circulating MIF concentrations in relation to breast cancer and a similar observation was made in another study using the MR approach [43]. A reason for this discrepancy could be a potential pleiotropic effect that MIF might have depending on its cellular localization and tumour stage and type [47]. It has been speculated that intracellular MIF in the breast cells has a protective function, whereas extracellular MIF, whether it is tumour-associated macrophage (TAM)-derived or produced by carcinoma cells upon stroma/tumour interactions, is pathogenic [47].

In our study, we also found an inverse association between genetically proxied circulating IL-1ra concentrations and endometrial cancer risk. Few prospective studies have evaluated IL-1ra in relation to endometrial cancer [51–53]. In a nested case-control study in the European Prospective Investigation into Cancer (EPIC), elevated concentrations of IL-1ra were associated with higher endometrial cancer risk [52]. It should be noted though that a large proportion of the measurements of IL-1ra (52%) was below the assay limit of detection. Another EPIC study, in the context of a factor analysis, provided evidence for a positive association between IL-1ra and endometrial cancer only in post-menopausal women, while adjustment for BMI markedly attenuated risk estimates [51]. A case-control study nested within the PLCO cohort reported null associations between IL-1ra and endometrial cancer risk [53]. Even though observational studies have shown that IL-1ra might act as a pro-inflammatory agent, mechanistic plausibility for a protective role of IL-1ra in cancer was demonstrated in experimental studies [54–56]. Little epidemiological and experimental evidence is currently available to support the observed positive association for MIG in relation to endometrial cancer, although associations with cancer outcomes other than endometrial have been reported [57].

A positive association was found between genetically proxied CTACK and lung cancer in never smokers and a nominally significant association in small-cell lung cancer. Findings from experimental studies have shown that CTACK is highly expressed in tumour cells with metastatic potential [58]. Additionally, a recent biomarker analysis using an antibody array demonstrated that 17 cytokines, among them CTACK, were differentially expressed in serum samples of non-small-cell lung cancer patients compared to healthy controls [59]. On the other hand, a nested case-control study within the PLCO, including 526 cases and 592 controls, was inconclusive on the association between pre-diagnostic plasma CTACK concentrations and lung cancer (Q4 versus Q1: OR = 0.93, 0.64 to 1.35) [57]. Our analysis also showed an inverse association between genetically proxied IL-18 concentrations and overall lung cancer and lung adenocarcinoma. Our observations are in line with findings from experimental research that has demonstrated an antitumour activity of IL-18 on lung cancer [60, 61]. It has been shown that IL-18 exhibits a variety of biological activities with implications in tumour initiation and development. IL-18 can activate T-helper cells, which produce cytokines that interact with activated natural killer cells and mediate the antitumour activity of IL-18. Furthermore, IL-18 has anti-angiogenic and pro-lymphangiogenesis properties, which contribute to its antitumour activity.

We also found inverse associations for RANTES in relation to invasive epithelial ovarian cancer and serous ovarian cancer. Such an inverse association is not supported by the limited to date evidence that largely comes from experimental studies and suggest that RANTES is positively associated with cancer stem-like cells differentiation and tumour angiogenesis, tumour immune tolerance and invasion, and chemoresistance [62–64]. On the other hand, a study that used publicly available microarray data-sets, deposited in the National Center for Biotechnology Information (NCBI) Gene Expression Omnibus (GEO) demonstrated that the association between RANTES and overall survival (OS) among ovarian cancer patients was dependent on the TP53 mutation status and higher expression of RANTES was associated with better OS only on TP53 mutant serous ovarian cancer [65].

Among the strengths of our analyses are the wide range of inflammatory cytokines that we covered, and the large sample size that was used in most of the analyses that we performed. Another strength is the approach that we used for instrument selection by using variants in close proximity to the encoding gene region, minimising the likelihood of horizontal pleiotropy [44]. Since cis-acting regulatory variants in the vicinity of genes influence mRNA and protein expression, and the majority of drug targets are proteins, an MR analysis using cis defined instruments is likely to have translational relevance [66]. In support of this notion, a number of studies during the last decade have demonstrated that variants in genes encoding a drug target mimic the effect of modifying the same target by use of pharmacological agents [67]. In addition, lifestyle changes such as changes in dietary habits, weight reduction and smoking cessation have been associated with changes in plasma concentrations of inflammatory biomarkers [68–70]. Considering that such changes can have a significant impact on the incidence of cancer when applied at the population scale, with minimal adverse effects, future studies that will aid in delineating such mediating effects are warranted. Our study’s primary limitation is the use of a single instrument or few instruments in some of the analyses, which may have affected power to reject the null hypothesis for some associations. The null findings for some associations are not necessarily indicative of the cytokines having no effect, since there were several cytokines with weak instruments. In addition, there may be non-linear effects, time-dependent effects or inflammation-environment interactions that are not captured by the current analysis. Moreover, potential synergistic effects between the studied cytokines and network cytokine approaches were not considered. Furthermore, since the number of independent SNPs required for the MR sensitivity analyses (i.e. weighted median, ConMix, MR Egger and PRESSO) to work properly is quite large, which is not the case in most of the analyses that we performed due to the cis-instrument definition approach, results of these analyses should be interpreted with caution. Another important point is that, although using the cis-eQTL definition (by retaining variants that are additionally associated with tissue specific expression), the cytokine expression component throughout the body (including in target organs) is partially captured, as measured cytokine concentrations in circulation may not relate to tissue expression. In addition, different parameters of gene expression, namely tissue specific and exposure specific expression are not accounted for in MR analyses. Even though we used a wide panel of inflammatory cytokines, genetic instruments were not available for several additional cytokines that may be implicated in cancer, such as IL-13, IFN-gamma and CXCL13. Future larger single- and multi-trait GWASs of cytokine concentrations, and MR studies with individual-level data could address some of the latter issues.

## Conclusions

In conclusion, we used novel instruments that incorporate gene expression relevance and large-scale genetic data for various cancer outcomes in MR analyses to investigate the associations of more than 30 circulating inflammatory cytokines with cancer risk. We reported several robust associations, though further validation is needed to assess the potential of these cytokines to be used as drug or lifestyle targets for cancer prevention.

## Supplementary Information

**Additional file 1.** Members from the PRACTICAL Consortium, CRUK, BPC3, CAPS and PEGASUS.

**Additional file 2.** (Supplemental Tables): Supplementary Table 1. Sources of GX instruments per cytokine & genetic locus of cytokine. Supplementary Table 2. Genetic association estimates that were used in the MR analyses. Supplementary Table 3. Sources of GY instruments per cancer outcome. Supplementary Table 4. Genetic association estimates that were used in the MR analyses. Supplementary Table 5. Summary of the MR results based on the cis-pQTL instrument definition. Supplementary Table 6. Summary of the MR results based on the cis-eQTL instrument definition. Supplementary Table 7. Identification of druggable targets using publicly available repositories. Supplementary Table 8. Summary of epidemiological evidence linking prediagnostic cytokine concentrations to cancer risk. Supplementary Table 9. KeGG pathways in which significant cytokines are involved. Supplementary Table 10. Colocalization analysis. Supplementary Table 11a. Secondary cytokines associated with selected instruments, based on the gwas included in the study. Supplementary Table 11b. Secondary traits associated with selected instruments, based on a phenoscanner search. Supplementary Table 12a. Sensitivity analyses excluding potentially pleiotropic genetic variants. Supplementary Table 12b. Sensitivity analyses accounting for LD among genetic variants. Supplementary Table 13. Replication of the significant associations in the UK Biobank.

**Additional file 3.** Supplemental Methods (Details on the Finnish GWAS, the meta-analysis with the SCALLOP or the INTERVAL GWAS and the Mendelian randomisation analyses).

**Additional file 4.** (Supplemental Figures): Supplementary figure 1. Correlation of MR-IVW estimates (betas) using the two different instrument definitions. Y axis represents the MR IVW estimates using the cis-eQTL and X axis represents the MR IVW estimates using the cis-pQTL instrument definition. Supplementary figure 2. Associations that showed MR evidence for both causality and colocalization (posterior probability>0.8) are plotted, within ±500 kb of the gene locus of the exposure cytokine.

## Data Availability

All data used in this work are presented in the Additional files that accompany the manuscript and are available in the original publications.

## Abbreviations

activePAI: Active plasminogen activator inhibitor
BCAC: Breast Cancer Association Consortium
bNGF: Beta nerve growth factor
CIMBA: Consortium of Investigators of Modifiers of BRCA1/2
cis-eQTL: cis-expression quantitative trait locus
cis-pQTL: cis-protein quantitative trait locus
ConMix: Contamination mixture
CRP: C-reactive protein
CTACK: Cutaneous T-cell attracting chemokine
E2C2: Epidemiology of Endometrial Cancer Consortium
ECAC: Endometrial Cancer Association Consortium
ER: Estrogen receptor
FDR: False discovery rate
FGFBasic: Basic fibroblast growth factor
GROa: Growth-regulated oncogene-alpha
GTEx: Genotype-tissue expression
GWAS: Genome-wide association study
HER: Epidermal growth factor receptor
HGF: Hepatocyte growth factor
IL: Interleukin
ILCCO: International Lung Cancer Consortium
IP-10: Interferon gamma-induced protein 10
IVW: Inverse-variance weighted
KEGG: Kyoto encyclopedia of genes and genomes
LD: Linkage disequilibrium
MAF: Minor allele frequency
MCP: Monocyte chemotactic protein
MCSF: Macrophage colony-stimulating factor
MeSH: Medical subject headings
MIF: Macrophage migration inhibitory factor
MIG: Monokine induced by interferon-gamma
MIP: Macrophage inflammatory protein
MR: Mendelian randomisation
MR-PRESSO: Mendelian randomisation Pleiotropy RESidual sum and outlier
NFBC: Northern Finland birth cohort
OCAC: Ovarian Cancer Association Consortium
OR: Odds ratio
PDGFbb: Platelet-derived growth factor BB
PR: Progesterone receptor
PRACTICAL: Prostate Cancer Association Group to Investigate Cancer-Associated Alterations in the Genome
SCF: Stem cell factor
SCGFb: Stem cell growth factor beta
SD: Standard deviation
SeSelectin: Soluble E-selectin
sICAM: Soluble intercellular adhesion molecule
SNPs: Single nucleotide polymorphisms
sVCAM: Soluble vascular cell adhesion molecule
TNF: Tumour necrosis factor
TRAIL: TNF-related apoptosis inducing ligand
TRICL: Transdisciplinary research of cancer in lung
UCSC: University of California Santa Cruz
VEGF: Vascular endothelial growth factor
