# Supplementary material for: Circulating inflammatory cytokines and risk of five cancers: a Mendelian randomization analysis
Source: BMC Med. 2022 Jan 11;20:3. doi: 10.1186/s12916-021-02193-0 (PMC8750876; doi:10.1186/s12916-021-02193-0)
Supplement: Supplementary file 3 — Additional file 3. Supplemental Methods (Details on the Finnish GWAS, the meta-analysis with the SCALLOP or the INTERVAL GWAS and the Mendelian randomisation analyses). [file 12916_2021_2193_MOESM3_ESM.pdf]

Supplementary Methods

Cytokine instrument source

We previously conducted a genome-wide association study (GWAS) of circulating levels of 47 inflammatory cytokines, using samples from up to 13,365 Finnish individuals from the Northern Finland Birth Cohort 1966 (NFBC1966), the Cardiovascular Risk in Young Finns (YFS) study, and FINRISK 1997 and 2002. The cytokines were selected based on availability to full raw data from the above studies. Details of these GWAS are presented below.

Finnish cohorts

Northern Finland Birth Cohort 1966

Northern Finland Birth Cohort 1966 (NFBC1966) recruited pregnant women with expected date of delivery between 1st January and 31st December 1966 [1, 2]. Overall, 12,055 mothers (over 96% of eligible women) were followed from pregnancy onwards, with 12,058 live-born children in the cohort. In the offspring 31-year data collection in 1997, all cohort members with known addresses in either the Northern Finland or Helsinki area were invited to a clinical examination. In total, data was received for 6,033 participants, and DNA was successfully extracted for 5753 participants from fasted blood samples. Cytokines were quantified from overnight fasting plasma samples using Bio-Rad's Bio-Plex 200 system (Bio-Rad Laboratories, California, USA) with Milliplex Human Chemokine/Cytokine and CVD/Cytokine kits (Cat# HCYTOMAG-60K-12 and Cat# SPR349; Millipore, St Charles, Missouri, USA) and Bio-Plex Manager Software V.4.3 as previously described [3, 4]. Genotyping was conducted using Illumina HumanCNV-370DUO Analysis BeadChip (Illumina, California, USA) and imputed using Haplotype Reference Consortium imputation reference panel.

The Cardiovascular Risk in Young Finns Study

The Cardiovascular Risk in Young Finns (YFS) is an ongoing follow-up study of 3,596 children and adolescents aged 3, 6, 9, 12, 15, or 18 years. The subjects were randomly chosen from five university cities and their rural surroundings using Finnish population register. The baseline survey was held in 1980 and subsequent follow-up visits involving all five centers have been arranged in 1983, 1986, 1989, 2001, 2007, 2011 and 2017. The latest follow-up included also children and parents of the original participants.

Genotyping have been performed using the blood samples drawn at 2001 follow-up visit. Genotyping was performed with custom-build Illumina 670K array. The custom content replaced some poor performing SNPs on the Human610 BeadChip and added more CNV content after which the customized chip shared 562,643 SNPs with Illumina Human610 chip. Genotyping was performed for 2,556 samples. Prior to imputation, samples and probes with high missingness were excluded (MIND>0.05 and GENO<0.05). To exclude poorly functioning probes, we excluded SNPs deviating from Hardy-Weinberg equilibrium (HWE $p<1\times10^{-6}$). To exclude related samples, we used $\hat{\pi}$ cut-off of 0.20. The pair with greater missingness was removed. After the QC steps, the data set included 2,443 individuals and 546,674 probes. The imputation was performed with IMPUTE2 software by using 1000 Genomes Phase 3 release as reference panel. After imputation, poorly imputed and rare variants (INF<0.7 and MAC<3) were removed.

Biorad's Bio-Plex Pro Human Cytokine 27-plex Assay and 21-plex Assay were used to quantify circulating concentrations of 48 cytokines from serum samples drawn at 2007 follow-up visit. The assays were performed according to manufacturer's instructions, except the beads, detection antibodies, and streptavidin-phycoerythrin conjugate were used with 50% lower concentrations than recommended. Only measures within cytokine-specific detection range were included. Depending on the cytokine, imputed genotypes and cytokine concentrations were available for 116 to 2,019 samples. GWAS were run with Snptest2 software.

FINRISK

FINRISK surveys are population-based cross-sectional studies which began in 1972. A new sample is recruited every five years to monitor the health status of Finnish population. Subset of individual-level data from 1992-2012 surveys is available through THL Biobank. Cytokine quantification for FINRISK1997 and FINRISK2002 samples was performed similarly as in YFS, but quantification was done using EDTA plasma in FINRISK1997 and heparin plasma in FINRISK2002. In FINRISK1997, a custom 20-plex array was used in cytokine quantification. Imputation was performed using 1000 Genomes Phase 3 as reference panel. Poorly imputed variants (INFO<0.7) and variants with low minor allele count (MAC<3) were excluded. Depending on the cytokine, imputed genotypes and cytokine concentrations were available for 3,440 to 4,613 samples from FINRISK1997 and 843 to 1,705 samples from FINRISK2002.

Cytokine genome-wide association study

We conducted GWAS for 41 cytokines in FINRISK+YFS population and for 16 cytokines in NFBC1966 8 (**see Supplementary Table 11 below**) [3]. The data pre-processing was done in a

similar manner to previous GWAS analyses [3, 5]. Inverse-normal rank transformation was first applied to the traits, before regressing the transformed measures on age, sex and the first 10 genetic principal components. In contrast to the previous analyses [3, 5] we did not add BMI as a covariate, as this could potentially introduce collider bias into consequent MR analyses [6]. The inverse-normal rank transformation was again applied to the residuals of this regression, and these transformed residual estimates were used as response variables in the GWAS. The GWAS was conducted in each study using an additive genetic model with SNPTEST2 software [7]. The results for variants which showed poor imputation quality (model info<0.7) or low minor allele frequency (MAF<0.05) were discarded. For the ten cytokines available in both NFBC1966 and FINRISK+YFS (see table below), the summary statistics were pooled by inverse variance weighted fixed-effects meta-analysis using Metal software [8].

Cytokines and their data sources:

		Sample size			Total sample size*
Cytokine	Abbreviation	NFBC1966	FINRISK	YFS	
Active plasminogen activator inhibitor-1	activePAI1	5199			5199
Beta nerve growth factor	βNGF		1620	1950	3531
Cutaneous T-cell attracting (CCL27)	CTACK		1651	2019	3631
Eotaxin (CCL11)	Eotaxin		6186	2011	8153
Basic fibroblast growth factor	FGFBasic		5592	2017	7565
Granulocyte colony-stimulating factor	GCSF		1544	2018	7904
Growth regulated oncogene-alpha (CXCL1)	GROα		1541	2003	3505
Hepatocyte growth factor	HGF		6317	2019	8292
Interferon-gamma	IFNγ		5726	2019	7701
Interleukin-10	IL10		5708	2016	7681
Interleukin-12p70	IL12p70		6295	2019	8270†
Interleukin-13	IL13		1577	2019	3557
Interleukin-16	IL16		1663	1858	3483
Interleukin-17	IL17	5071	5785	2019	12831

Interleukin-18	IL18		1656	2019	3636
Interleukin-1-alpha	IL1α	5014			5014
Interleukin-1-beta	IL1β	5067	1330	2018	8376
Interleukin-1 receptor antagonist	IL1ra	4957	1658	2019	8595
Interleukin-2	IL2		1498	2016	3475
Interleukin-2 receptor, alpha subunit	IL2rα		1704	2012	3677
Interleukin-4	IL4	5059	6149	2019	13183
Interleukin-5	IL5		1386	2017	3364
Interleukin-6	IL6	5063	6215	2018	13252
Interleukin-7	IL7		1429	2019	3409
Interleukin-8 (CXCL8)	IL8	5071	1546	2019	8597
Interleukin-9	IL9		1656	2017	3634
Interferon gamma-induced protein 10 (CXCL10)	IP10	5072	1705	2019	8757
Monocyte chemotactic protein-1 (CCL2)	MCP1	5072	6318	2019	13365
Monocyte specific chemokine 3 (CCL7)	MCP3		843	256	843
Macrophage colony-stimulating factor	MCSF		1632	866	840
Macrophage migration inhibitory factor (glycosylation-inhibiting factor)	MIF		1516	2017	3494
Monokine induced by interferon-gamma (CXCL9)	MIG		1705	2019	3685
Macrophage inflammatory protein-1a (CCL3)	MIP1α		1542	2019	3522
Macrophage inflammatory protein-1b (CCL4)	MIP1β		6268	2019	8243
Platelet derived growth factor BB	PDGFbb		6318	2019	8293
Regulated on Activation, Normal T Cell Expressed and Secreted (CCL5)	RANTES		1585	1869	3421
Soluble CD40 ligand	sCD40L	5067			5067

Stem cell factor	SCF		6316	2018	8290
Stem cell growth factor beta	SCGFβ		1704	2017	3682
Stromal cell-derived factor-1 alpha (CXCL12)	SDF1α		6003	1826	5998
soluble E-selectin	sE-selectin	5199			5199
soluble intercellular adhesion molecule-1	sICAM1	5199			5199
soluble vascular cell adhesion molecule 1	sVCAM1	5199			5199
Tumor necrosis factor-alpha	TNFα	5068	1474	2019	8522
Tumor necrosis factor-beta	TNFβ		1450	116	1559
TNF-related apoptosis inducing ligand	TRAIL		6218	2012	8186
Vascular endothelial growth factor	VEGF	5037	5143	2019	12155

*The total sample size with full genomic and cytokine data after quality control.

NFBC1966 = Northern Finland Birth Cohort 1966; YFS = Young Finns Study; FINRISK = FINRISK Study.

Meta-analysis of Finnish GWAS and additional publicly available sources

Publicly available data for several cytokines (common to the 41 inflammatory cytokines for which we performed GWAS as described above) were available from two additional sources: a GWAS of up to 3,301 individuals of European descent from the INTERVAL study and a GWAS of up to 21,758 individuals of European descent from the SCALLOP consortium [9, 10]. In order to obtain the most robust estimates for any given cytokine, the associations of single nucleotide polymorphisms (SNPs) with inflammatory cytokines from (any of) these sources were pooled with the Finnish GWAS estimates, when estimates between GWAS correlated well, as described below. At an exploratory stage we examined the correlation between the beta coefficients of the same SNP-Cytokine pair from the INTERVAL and Finnish GWAS, focusing on SNPs with $r^2<0.1$ and associations with $p<10^{-5}$ in at least one of the two GWAS, by

performing linear regression for the beta coefficients from INTERVAL GWAS against the beta coefficients from the Finnish GWAS. We performed the same analysis to examine the correlation of cytokines that were common to the Finnish and the SCALLOP GWAS. Estimates were considered to correlate well when P-value for correlation was not greater than 0.05. In case of good correlation we first converted the original GWAS into the same scale as Finnish using the intercept and beta coefficients from the linear regression, and then pooled the estimates from the corresponding studies, by fixed-effects meta-analysis, weighing by standard error. We did not meta-analyse all three sources together due to the overlap between SCALLOP and INTERVAL for most of the cytokines of interest (the SCALLOP GWAS contains the INTERVAL study).

Mendelian randomization

The selected genetic variants, in order to be valid instruments for the MR analysis, must meet the following criteria: (i) they should be strongly associated with the circulating concentrations of the cytokine, (ii) they should be independent of any potential confounding variable of the cytokine-cancer association and (iii) they should affect cancer only through the cytokine being instrumented. The presence of horizontal pleiotropy, that occurs when a variant influences the outcome through other traits (pathways) that bypass the exposure of interest, is the most common reason for violation of the third assumption. To explore the robustness of our findings to potential pleiotropic effects of the variants, we applied several sensitivity analyses. These were the weighted-median, contamination mixture (ConMix), MR-Egger and MR-PRESSO analyses.

The **weighted-median** approach orders the MR estimates from each genetic instrument by their magnitude weighted for their precision and produces an overall MR estimate based on the median value [11]. It can provide a consistent estimate of the causal effect even when up to 50% of the weight comes from instruments that are not valid.

The **ConMix model**, uses a likelihood-based approach using the variant-specific causal estimates [12]. Under the assumption that there is a single causal effect of the risk factor on the outcome, the ConMix model can estimate this effect robustly and efficiently, even in the presence of some invalid genetic variants. Additionally, in the presence of many variants, ConMix can identify subgroups of genetic variants having mutually similar causal estimates. Identification of such distinct groups suggests that there may be several causal mechanisms associated with the same risk factor that affect the outcome to different degrees.

The **MR-Egger** approach regresses variant-outcome estimates on variant-exposure estimates weighted by the precision of the variant-outcome associations. The regression slope provides an estimate of the causal effect, even when all the variants are invalid due to violation of the third MR assumption [13]. For the causal effect estimate to be valid, the method assumes that the distribution of direct effects of candidate instruments on the outcome is independent from the distribution of associations with the risk factor, known as the Instrument Strength Independent of Direct Effect (InSIDE) assumption. The intercept from the regression can be interpreted as an estimate of the average pleiotropic effect across the genetic variants.

The Mendelian randomization Pleiotropy RESidual Sum and Outlier (**MR-PRESSO**) detects outlying variants based on the regression representation of the inverse-variance weighted method, and repeats MR analyses after excluding any identified outlier variants [14]. MR-PRESSO assumes that at least 50% of the genetic variants be valid instruments, have balanced pleiotropy and that the InSIDE assumption holds.

References

1. Rantakallio, P., *Groups at risk in low birth weight infants and perinatal mortality.* Acta Paediatr Scand, 1969. **193**: p. Suppl 193:1+.
2. *University of Oulu. Northern Finland Birth Cohort 1966. http://urn.fi/urn:nbn:fi:att:bc1e5408-980e-4a62-b899-43bec3755243*.
3. Sliz, E., et al., *Genome-wide association study identifies seven novel loci associating with circulating cytokines and cell adhesion molecules in Finns.* J Med Genet, 2019. **56**(9): p. 607-616.
4. Saukkonen, T., et al., *Adipokines and inflammatory markers in elderly subjects with high risk of type 2 diabetes and cardiovascular disease.* Sci Rep, 2018. **8**(1): p. 12816.
5. Ahola-Olli, A.V., et al., *Genome-wide Association Study Identifies 27 Loci Influencing Concentrations of Circulating Cytokines and Growth Factors.* Am J Hum Genet, 2017. **100**(1): p. 40-50.
6. Day, F.R., et al., *A Robust Example of Collider Bias in a Genetic Association Study.* Am J Hum Genet, 2016. **98**(2): p. 392-3.
7. Marchini, J., et al., *A new multipoint method for genome-wide association studies by imputation of genotypes.* Nat Genet, 2007. **39**(7): p. 906-13.
8. Willer, C.J., Y. Li, and G.R. Abecasis, *METAL: fast and efficient meta-analysis of genomewide association scans.* Bioinformatics, 2010. **26**(17): p. 2190-1.
9. Folkersen, L., et al., *Genomic and drug target evaluation of 90 cardiovascular proteins in 30,931 individuals.* Nat Metab, 2020. **2**(10): p. 1135-1148.
10. Sun, B.B., et al., *Genomic atlas of the human plasma proteome.* Nature, 2018. **558**(7708): p. 73-79.
11. Bowden, J., et al., *Consistent Estimation in Mendelian Randomization with Some Invalid Instruments Using a Weighted Median Estimator.* Genet Epidemiol, 2016. **40**(4): p. 304-14.
12. Burgess, S., et al., *A robust and efficient method for Mendelian randomization with hundreds of genetic variants.* Nat Commun, 2020. **11**(1): p. 376.
13. Bowden, J., G. Davey Smith, and S. Burgess, *Mendelian randomization with invalid instruments: effect estimation and bias detection through Egger regression.* Int J Epidemiol, 2015. **44**(2): p. 512-25.
14. Verbanck, M., et al., *Detection of widespread horizontal pleiotropy in causal relationships inferred from Mendelian randomization between complex traits and diseases.* Nat Genet, 2018. **50**(5): p. 693-698.
