# Supplementary material for: Circulating inflammatory cytokines and risk of five cancers: a Mendelian randomization analysis
Source: BMC Med. 2022 Jan 11;20:3. doi: 10.1186/s12916-021-02193-0 (PMC8750876; doi:10.1186/s12916-021-02193-0)
Supplement: Supplementary file 4 — Additional file 4. (Supplemental Figures): Supplementary figure 1. Correlation of MR-IVW estimates (betas) using the two different instrument definitions. Y axis represents the MR IVW estimates using the cis-eQTL and X axis represents the MR IVW estimates using the cis-pQTL instrument definition. Supplementary figure 2. Associations that showed MR evidence for both causality and colocalization (posterior probability>0.8) are plotted, within ±500 kb of the gene locus of the exposure cytokine. [file 12916_2021_2193_MOESM4_ESM.pdf]

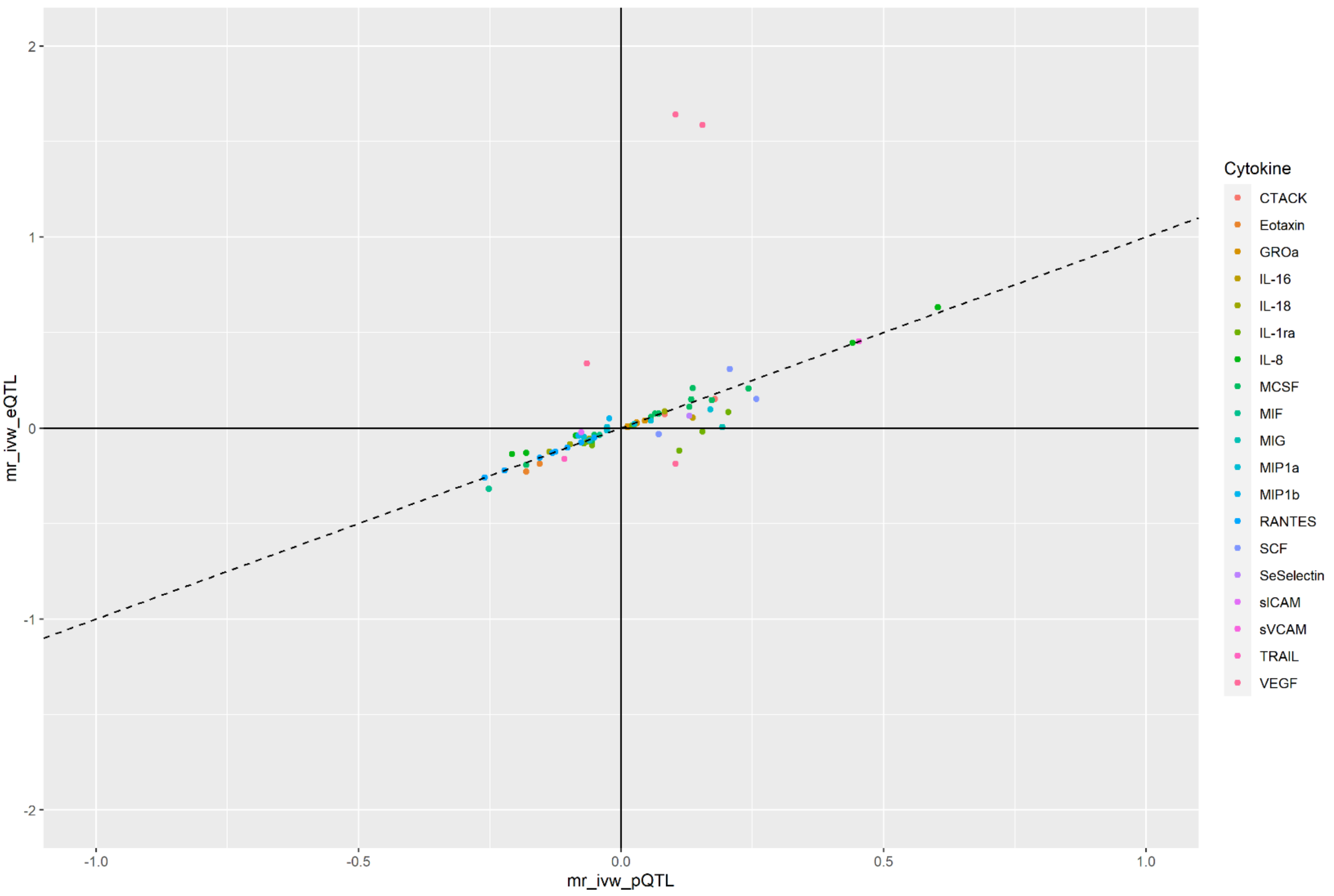

Supplementary figure 1. Correlation of MR-IVW estimates (betas) using the two different instrument definitions. Y axis represents the MR IVW estimates using the cis-eQTL and X axis represents the MR IVW estimates using the cis-pQTL instrument definition.

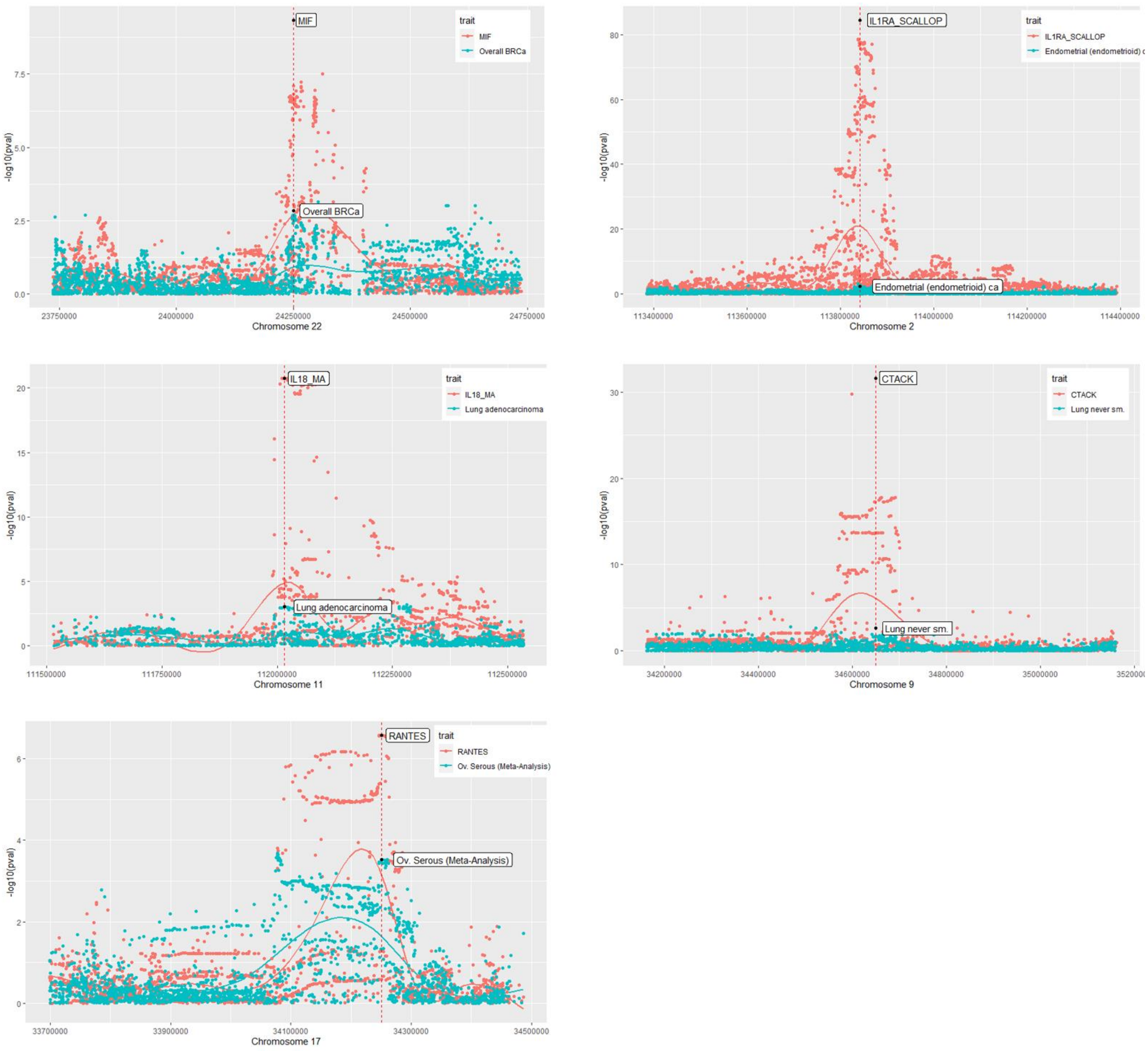

Supplementary figure 2. Associations that showed MR evidence for both causality and colocalization (posterior probability>0.8) are plotted, within ±500 kb of the gene locus of the exposure cytokine.
